# Novel carbon quantum dots from egg yolk oil and their haemostatic effects

**DOI:** 10.1038/s41598-017-04073-1

**Published:** 2017-06-30

**Authors:** Yan Zhao, Yue Zhang, Xiaoman Liu, Hui Kong, Yongzhi Wang, Gaofeng Qin, Peng Cao, Xingxing Song, Xin Yan, Qingguo Wang, Huihua Qu

**Affiliations:** 1 School of Basic Medical Sciences, Beijing University of Chinese Medicine, Chaoyang, Qu China; 2 School of Chinese Materia Medica, Beijing University of Chinese Medicine, Chaoyang, Qu China; 3 Center of Scientific Experiment, Beijing University of Chinese Medicine, Chaoyang, Qu China

## Abstract

In this study, the properties of egg yolk oil (EYO) were investigated. Water extraction, dialysis, and ultrafiltration were used to extract and purify EYO, and microscopy, spectrophotometry, and chromatography were used to identify carbon dots (CDs) present in EYO (EYO CDs). Morphology analyses demonstrated that CDs were almost spherical, with an average size of <10 nm, a lattice spacing of 0.267 nm, and a composition of mainly C, O, and Fe. The solution showed bright blue fluorescence at 365 nm. Tail haemorrhaging and liver haemorrhaging experiments showed that CD-treated mice had significantly shorter bleeding times than did control mice. Coagulation assays suggested that EYO CDs stimulate the intrinsic blood coagulation system and activate the fibrinogen system. Thus, EYO CDs possess the ability to activate haemostasis, which may lead to further investigations of this ingredient of traditional Chinese medicine.

## Introduction

Egg yolk oil (EYO) is obtained by refining cooked egg yolks of *Gallus gallus domesticus Brisson*. It has been used as a traditional medicine in China for more than a thousand years. The first record of EYO treatment for burns and scalds was found in “*Set Prescription*”, dated around 500 AD, and it was also recorded in the famous “*Compendium of Materia Medica*” written during the Ming Dynasty^1^. Currently, EYO is widely used to treat all kinds of burn wounds^2^, as well as acute and chronic eczema in clinics^3^. Modern pharmacological studies have shown that EYO has analgesic, anti-oxidative, and anti-aging properties, and enhances memory, reduces blood fat, and improves microcirculation and oedema^1^.

**Figure 1:**
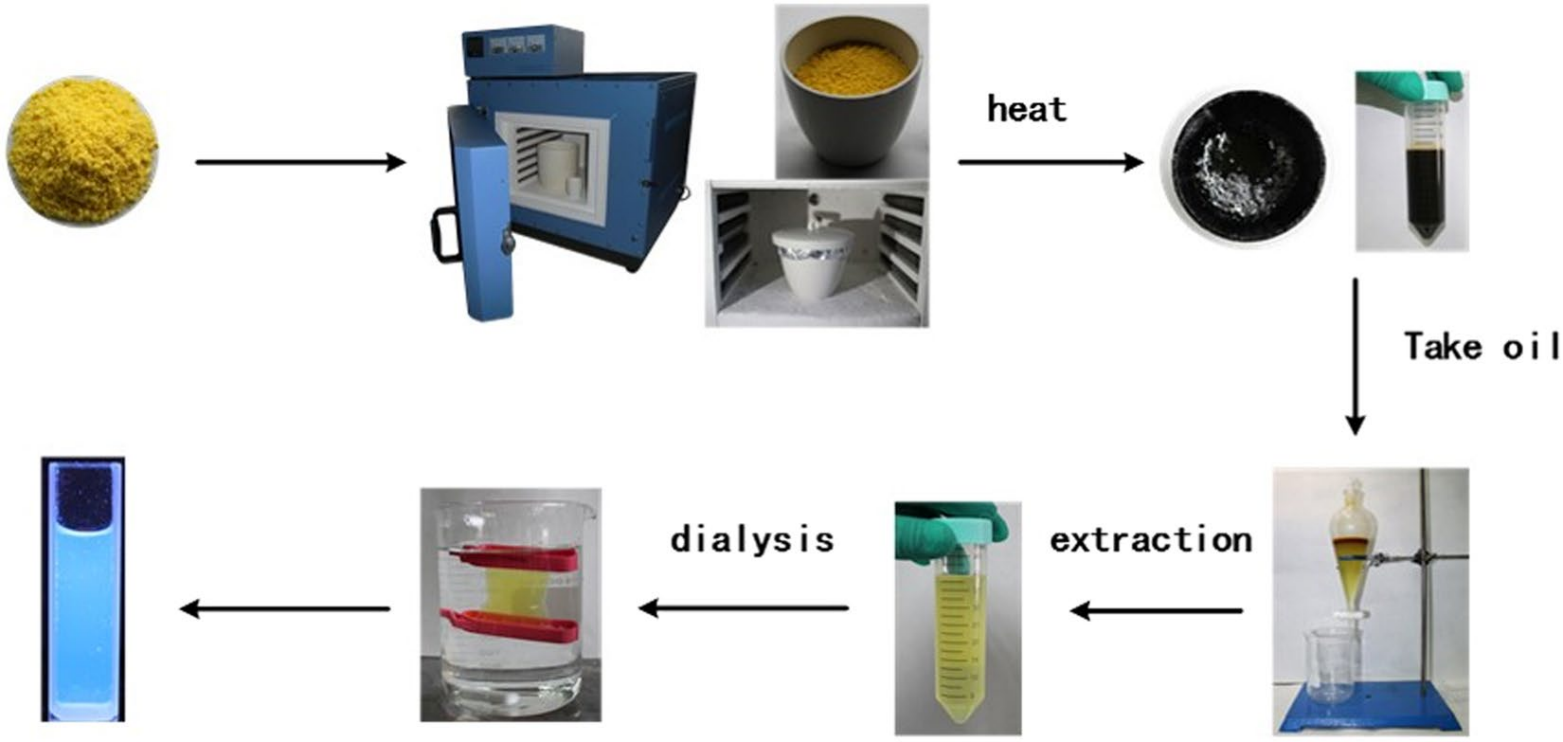
Preparation procedure of egg yolk oil carbon dots (EYO CDs).

The traditional preparation method for EYO involves heating cooked egg yolks first under a low flame prior to water evaporation, and then under a high flame until oil flow is induced (Fig. 1). Natural EYO is black and unstable, with an unpleasant odour and a low yield; many methods for EYO extraction have been developed, including dry distillation, baking, reduced pressure distillation, solvent extraction, supercritical CO_2_ extraction, sub-critical propane extraction, and enzyme-based processes^3^.

Interestingly, the synthetic method of EYO is similar to the formation of carbon dots. Carbon dots (CDs), one of the most promising fluorescent nanocomposite, have become the hot research topics because of their novel properties such as unique photoluminescence^4^, high resistance to photobleaching, high aqueous solubility^5^, high biocompatibility and low toxicity^6^.

The synthetic procedures for CDs can be divided into two main approaches: bottom-up and top-down synthesis^4, 5^. The bottom-up route builds nanostructures from small organic molecular precursors by pyrolysis, combustion or hydrothermal methods, which include electrochemical oxidation, exfoliation of carbon soot laser ablation. While the top-down approach is based on cutting small sheets via physical or chemical techniques until the demanded particle size is reached which include pyrolysis, microwave-assisted approach hydrothermal method doping with heteroatoms such as nitrogen and phosphor, or metals such as Au improves the electrical conductivity and solubility of CDs^5, 7^. Current trend has been focused on the preparation of CDs from “green” materials as a carbon source, during which milk^7^, juice^8^, eggshell^6^ even food waste^9^ can be used.

This suggests the synthetic method of EYO and production approach of CDs were in similar. Then here comes the question: is there CDs exist in the EYO? To circumvent this, we extracted EYO using pure water and investigated the water-soluble components. We found that the water extract showed strong fluorescence; therefore, we purified it and identified the active ingredients via fluorescence spectroscopy and transmission electron microscopy. We found carbon dots (CDs) in the resulting purified solution, and thus investigated the properties and active effects of these CDs.

## Methods

### Chemicals and materials

EYO was obtained from Beijing Aoboxing Bio-tech Co., Ltd (Beijing, China). Haemocoagulase for injection was obtained from Jinzhou Ahon Pharmaceutical Co., Ltd (Liaoning, China). Ethyl acetate and other analytical-grade chemical reagents were obtained from Sinopharm Chemical Reagents Beijing (Beijing, China). Dialysis bags of 1,000 Da molecular weight cutoff (MWCO) were purchased from Beijing Ruida Henghui Technology Development Co., Ltd (Beijing, China). Ultrafiltration tubes of 10 kDa cutoff were acquired from Sartorius Instruments Co., Ltd (Beijing, China).

### Animals

This study was performed in accordance with the Guide for the Care and Use of Laboratory Animals and was approved by the Committee of Ethics of Animal Experimentation of the Beijing University of Chinese Medicine. Male Kunming mice, weighing 32.0 ± 1.1 g, were purchased from the Laboratory Animal Center, Si Beifu Laboratory Animal Certificate of Conformity (temperature: 24.0 ± 1.0 °C, relative humidity: 55–65% and 12 h light/12 h dark cycle) and had *ad libitum* access to food and water. Animals were acclimatized to laboratory conditions for 1 week prior to experimentation and were fasted overnight before drug administration.

### Instrumentation

The EYO carbon quantum dots (EYO CDs) were produced in a digital muffle furnace from Beijing Zhongke Aobe Technology Co., Ltd (Beijing, China). Transmission electron microscopy (TEM) Images of EYO CDs were taken using a JEN-1230 electron microscope at an accelerating voltage of 100 kV (Japan Electron Optics Laboratory) and a Tecnai G2 20 TEM (FEI Company, USA) at an accelerating voltage of 200 kV. Fluorescence images were acquired using an OLYMPUS IX73 fluorescent microscope (Tokyo, Japan). Pure water was produced using a Great Wall Scientific Industry circulating water-type multi-purpose vacuum pump (Henan, China). The UV-Vis spectroscopy was performed using a CECIL instruments spectrophotometer (Cambridge, United Kingdom). Fourier transform infrared (FTIR) spectroscopy was performed using a Thermo spectrometer (California, USA).

### Preparation of egg yolk oil

Sixty grams of chicken egg yolk powder was weighed and placed into a crucible, which was covered with aluminium foil, the lid was closed, and the crucible was placed into the muffle furnace. The temperature was then increased to 260 °C over an hour, maintained for another hour, and then cooled to room temperature. EYO (about 25 mL or 25 g) was extracted from the crucible bottom.

### Preparation of EYO CDs

Egg yolk powder was extracted by water three times. The aqueous solutions were pooled and concentrated to the original volume of EYO. The concentrated solution was purified using a 1 kDa MWCO dialysis membrane against water for 3 days, with water changes every 4 h. The retentate was then passed through a 10 kDa cutoff ultrafiltration tube. The final solution, containing the EYO CDs (concentration 28.6 mg/mL), was stored at 4 °C until experimentation.

### Identification of EYO CDs

Transmission electron microscopy (TEM) was used to identify and characterize EYO CDs. A 5 µL aliquot of diluted EYO CD stock solution was placed on a 200-mesh copper grid and maintained for 8 h until the water had completely evaporated. A TEM microscope operating at 100 kV was used to view the morphology and size distribution of anhydrous EYO CDs. High-resolution transmission electron microscopy (HRTEM) images were taken using an electron microscope at an accelerating voltage of 200 kV to view detailed morphology.

An FTIR spectrometer was employed to analyse the surface chemical bonds of EYO CDs. UV-visible and fluorescence spectroscopy were employed to investigate the optical properties of the EYO CDs, with water used as a blank control.

Thin-layer chromatography (TLC) was employed to identify the possible compounds in the water extract. Ethyl acetate was used as the mobile phase in TLC, and the resulting spots were visualized at 365 nm.

### Quantum yield determination

The quantum yield (F) of the EYO CDs and the conjugate was measured by comparing the integrated photoluminescence intensities and the absorbency values with those of the reference, quinine sulphate. The EYO CDs were dissolved in double distilled water and quinine sulphate was dissolved in 0.1 M H_2_SO_4_. The quantum yield of EYO CDs was determined as previously reported by Hu *et al*.^10^.

### Haemostatic effects of EYO CDs

Forty-five Kunming male mice were randomly assigned to five experimental groups (n = 9 per group). The groups were termed normal (treated with saline), control (treated with haemocoagulase), high-dose EYO CDs (223 mg/kg), medium-dose EYO CDs (112 mg/kg), and low-dose EYO CDs (56 mg/kg). Mouse tail-bleeding time was measured using a modification of a previously described technique^11^. Briefly, mice were anaesthetized using 10% chloral hydrate and placed into a cylinder prior to drug intraperitoneal injection. The tail was pulled through the cylinder bottom, laid flat on the platform, and then transected with a sterile scalpel at a point where the tail diameter was approximately 1.11 mm (10 mm from the tip). After transection, the tail was immediately placed on filter paper, and the time to bleeding cessation was recorded. The tail was blotted with filter paper every 30 s to absorb excess blood.

Kunming male mice were fed for 2 weeks after transection and evaluated for liver haemostatic effects^12^. Grouping and dosing were consistent with those of the tail transection experiment. Mice were administered drugs via intraperitoneal injection 1.5 h before anaesthesia. At 30 min after anaesthesia, the left lobe of the liver was punctured with a 2-mL syringe, generating a 2-mm deep wound, 0.5 cm from the liver edge. Bleeding time was recorded as the time between the puncture and the time at which blood no longer stained the filter paper. At the end of the experiment and prior to recovery from anaesthesia, mice were euthanized by cervical dislocation.

### Haemostatic mechanism of EYO CDs

Forty-five healthy, male Kunming mice were used in the study. The mice were equally divided into a normal group (treated with saline, NS), positive drug group (treated with haemocoagulase, HC), high-dose EYO CDs (223 mg/kg), medium-dose EYO CDs (112 mg/kg), and low-dose EYO CDs (56 mg/kg). After removing an eyeball, blood was collected in 3.2% sodium citrate (the ratio of sodium citrate to plasma was 1: 9) and EDTA blood collection tubes, with gentle mixing for at least 5 min at room temperature before analysis. The blood was then analysed by routinely used and specific coagulation assays^13, 14^. The values of activated partial thromboplastin time (APTT), prothrombin time (PT), thrombin time (TT), fibrinogen (FIB) and platelets value (PLT) were measured.

### Statistical analyses

If data were normally distributed and the variances were equal, mean ± standard deviation was used for statistical description. Within-group differences were assessed with one-way ANOVA. If P < 0.05, Fisher’s Least Significant Difference method was used for multiple comparison testing. Data were plotted as bar graphs.

If data were not normally distributed or had unequal variances, medians and interquartile ranges were used for statistical description. Within-group differences were assessed with a nonparametric test. If P < 0.05, the Wilcoxon Signed-Rank Test was used to compare two groups, using the Bonferroni method to correct the P value. Data were shown as a box plot. P < 0.05 was considered statistically significant. Both *p < 0.05 and **p < 0.01 were used in pairwise comparisons.

## Results

### Characterization of EYO CDs

**Figure 2:**
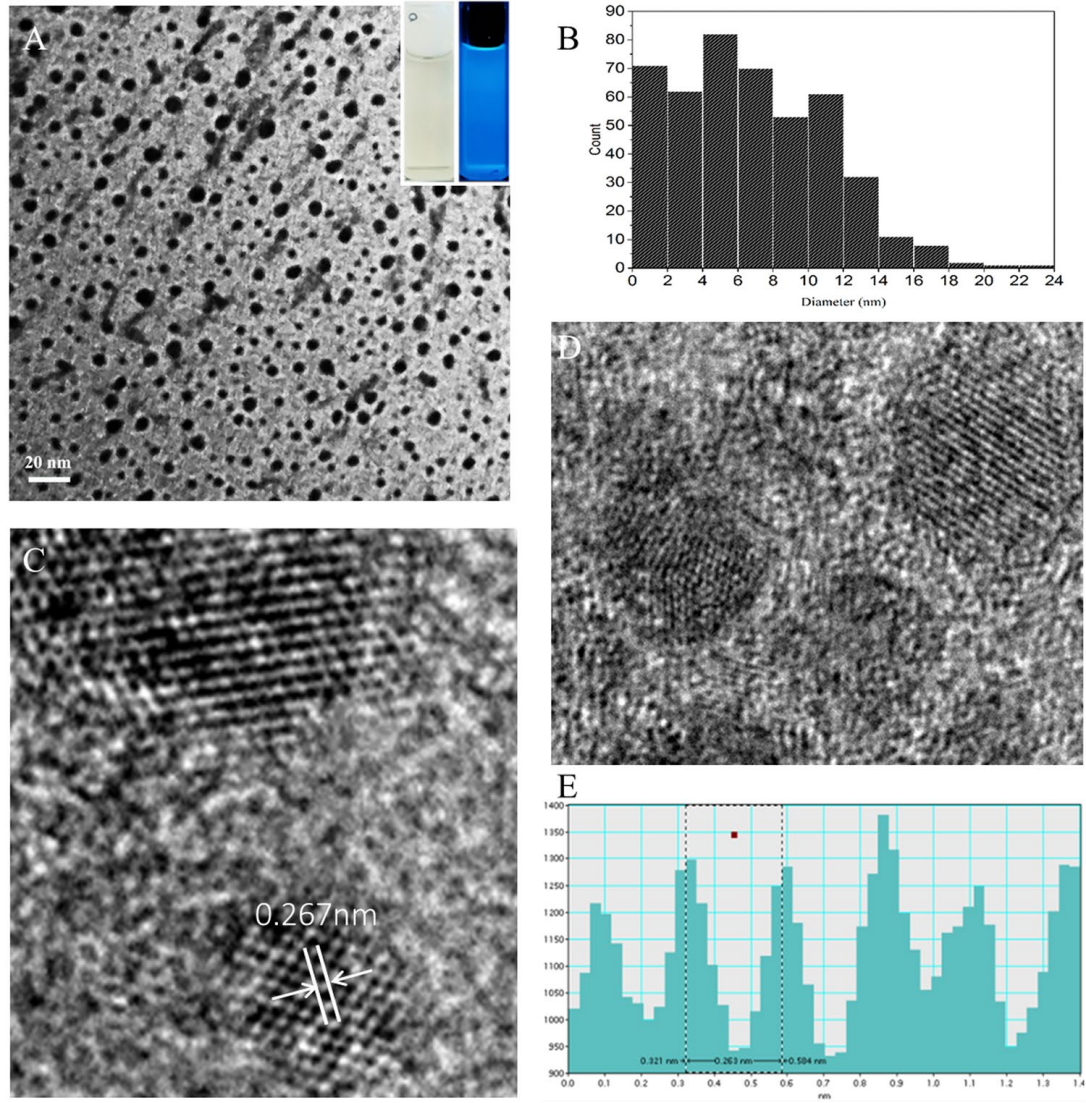
(**A**) Transmission electron microscopy (TEM) image of egg yolk oil carbon quantum dots (EYO CDs). (magnification 145,000x), the accelerating voltage was 100 kV. (**B**) Histograms of particle size distribution of EYO CDs. (magnification 880,000x), the accelerating voltage was 200 kV. (**C** and **D**) High-resolution TEM (HRTEM) images of individual EYO CDs. (**E**) The line profiles of the corresponding HRTEM images of EYO CDs. The lattice fringe (d = 0.267 nm) induced from the inplane diffraction of graphene.

TEM images (Fig. 2A) showed that CDs were nearly spherical, their average size was less than 10 nm (Fig. 2B)^15^.HRTEM images were showed in Fig. 2C,D, and their lattice spacing of EYO CDs was 0.267 nm (Fig. 2E). These morphology results were in accordance with those reported in previous studies^5, 16^.

**Figure 3:**
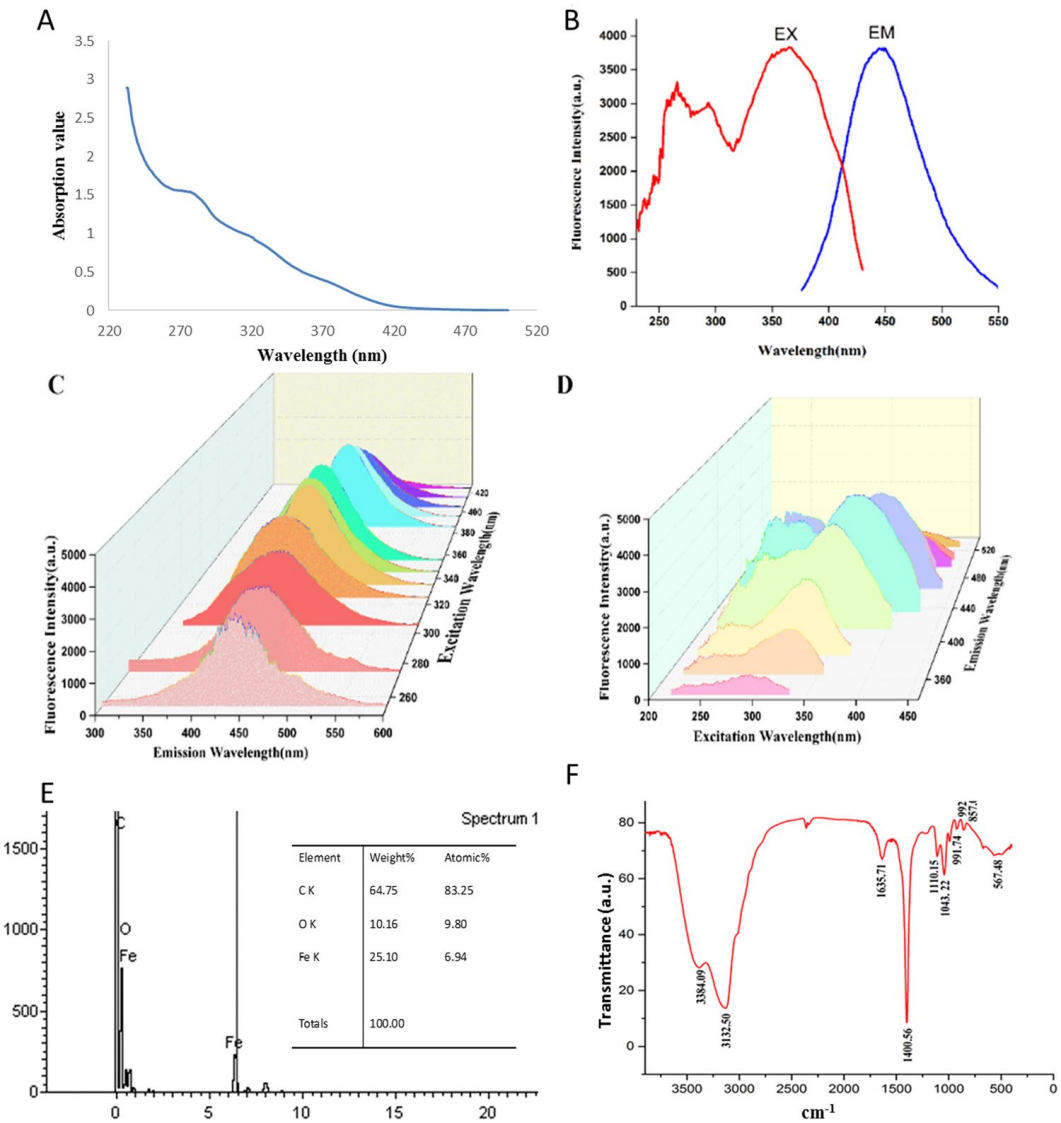
Characterization of egg yolk oil carbon quantum dots (EYO CDs). (**A**) UV absorption spectra of the EYO CDs at a concentration of 25 μg/mL. (**B**) Emission spectra of EYO CDs excited at 360 nm. (**C**) Fluorescence spectra of EYO CDs with different excitation wavelengths. (**D**) Excitation spectra of EYO CDs with different emission wavelengths. (**E**) Elemental analysis of EYO CDs. (**F**) Fourier transform infrared (FTIR) spectrum of the EYO CDs (32 scans at 2 cm^−1^ resolution in the scanning range of 400–4000 cm^−1^).

The UV spectrum of the EYO CDs is shown in Fig. 3A. The UV–Vis spectrum of the CDs shows a clear adsorption peak at 270 nm, which can be ascribed to the π–π* transition of the aromatic C=C bond. The n–π* transition of the heteroatom (such as S and N) doped surface state region was not observed^17^. The UV spectrum of benzo(α)pyrene showed a characteristic adsorption peak at approximately 294 nm^18^, which marked an obvious contrast to the EYO CD adsorption spectrum, indicating that there was no benzo(α)pyrene in the prepared EYO CD solution.

Figure 3B,C,D showed the fluorescence spectra of the EYO CDs. When the excitation wavelength changed from 200 to 400 nm, the maximum emission wavelength of the EYO CDs remained almost constant, with the same peak centred at 420 nm. While the emission wavelength changed from 200 to 400 nm, the maximum excitation wavelength changed from 300 to 440 nm. In this study, the quantum yield of EYO CDs was determined to be 5.01%.

Elemental analysis (Fig. 3E) by Tecnai G2 20 and transmission electron microscopy showed that three elements, C, O, and Fe, were present in EYO CDs, which was quite different from a previous report of amphiphilic egg-derived carbon dots^19^. The previous study generated the dots via electrode (voltage = 50 V, current = 2.4A)-irradiated raw eggs, and showed that they contained C, N, and O elements. This discrepancy may arise owing to differences in the preparation process.

The FTIR spectrum of the EYO CDs (Fig. 3F) revealed the presence of O-H groups at 3364 cm^−1^ ^20^ and C-H groups at 3132 cm^−1^ ^21^. The peaks at 1635 cm^−1^ and 1400 cm^−1^ were identified as COO^−^ groups, and the peaks at 1110 cm^−1^ and 1043 cm^−1^ ^10^ were attributed to C-O-C bonds, which imply that there are sp3 hybrid carbons with some sp2 carbons in CDs. The enhancement of the band at 567 cm^−1^ observed in the spectra was assigned to the Fe-O-O stretching mode ν (Fe–O_2_)^22^. Therefore, we concluded that the EYO CDs are mainly composed of sp2 graphitic carbons with sp3 carbon and abundant hydroxyl and carbonyl/carboxylate groups at their surfaces.

TLC showed that the EYO CD solution after dialysis (the solution in the dialysis bag or retentate) had no fluorescence spots along the solvent direction except for the original point, but the EYO CD dialysis solution (the solution outside of the dialysis bag or dialysate) and the EYO CD solution prior to dialysis (the original aqueous solution before dialysis) presented similar fluorescent spot patterns (Fig. S1). Those spots are likely to be indicative of small molecules such as amino acids, thus illustrating that materials with molecular weights smaller than 1 kDa were dialysed completely, and that the material in the dialysis bag had good purity. In this study, two dialysis steps were used to prepare CD solutions, with the first step using a 1 kDa MWCO dialysis bag and the second utilizing ultrafiltration through a 10 kDa ultrafiltration tube, in order to remove protein macromolecules. Our results indicate that this method is suitable for quantum dot purification.

### Haemostatic effects of EYO CDs

**Figure 4:**
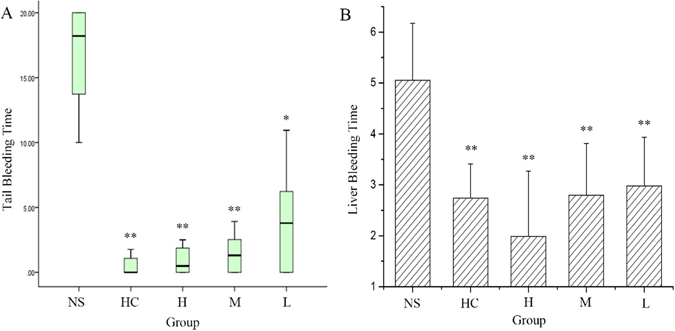
(**A**) Haemostatic effect of egg yolk oil carbon quantum dots (EYO CDs) in mouse tail transection model. (**B**) Haemostatic effect of EYO CDs evaluated by liver laceration bleeding model. The male Kunming mice were divided into normal group (treated with saline, NS), positive drug group (treated with haemocoagulase, HC), high-dose EYO CDs, medium-dose EYO CDs, and low-dose EYO CDs. (n = 9 per group).

We used two different methods to assess bleeding duration in mice. In the first test, mouse tails were transected and a filter paper was used to assess and quantify the blood oozing from the wound without disturbing clot formation. The differences in bleeding among normal (untreated), positive drug-treated, and EYO-CDs-treated mice were readily apparent from visual inspection of the filters (Fig. S2). The Fig. 4A showed that bleeding time in medium- and high-dose EYO-CDs-treated mice was similar to that of positive drug-treated mice and was remarkably lower than that in normal mice (P < 0.01). The bleeding time in the low-dose EYO CDs-treated mice was less than that of the normal group (P < 0.05) but higher than that of the positive control group.

A mouse liver laceration bleeding model was used for the *in vivo* examination of the haemostasis effect. Figure 4B shows the time required to achieve haemostasis in this model. The mean bleeding times achieved were 2.07 ± 1.41 min for high-dose EYO CDs-treated, 2.80 ± 1.01 min for medium-dose EYO CDs-treated, 2.98 ± 0.96 min for low-dose EYO CDs-treated, 2.37 ± 0.99 min for positive controls, and 8.38 ± 1.33 min for untreated animals. All treated groups showed significantly decreased bleeding times compared to that reported for the untreated group (P < 0.05). There were no differences between the high dose, medium dose, low dose, and positive control groups. Importantly, the tail and liver bleeding results show that EYO CDs have a certain dose-dependent haemostatic effect when administered in the range of 56–223 mg/kg.

### Haemostatic mechanism of EYO CDs

**Figure 5:**
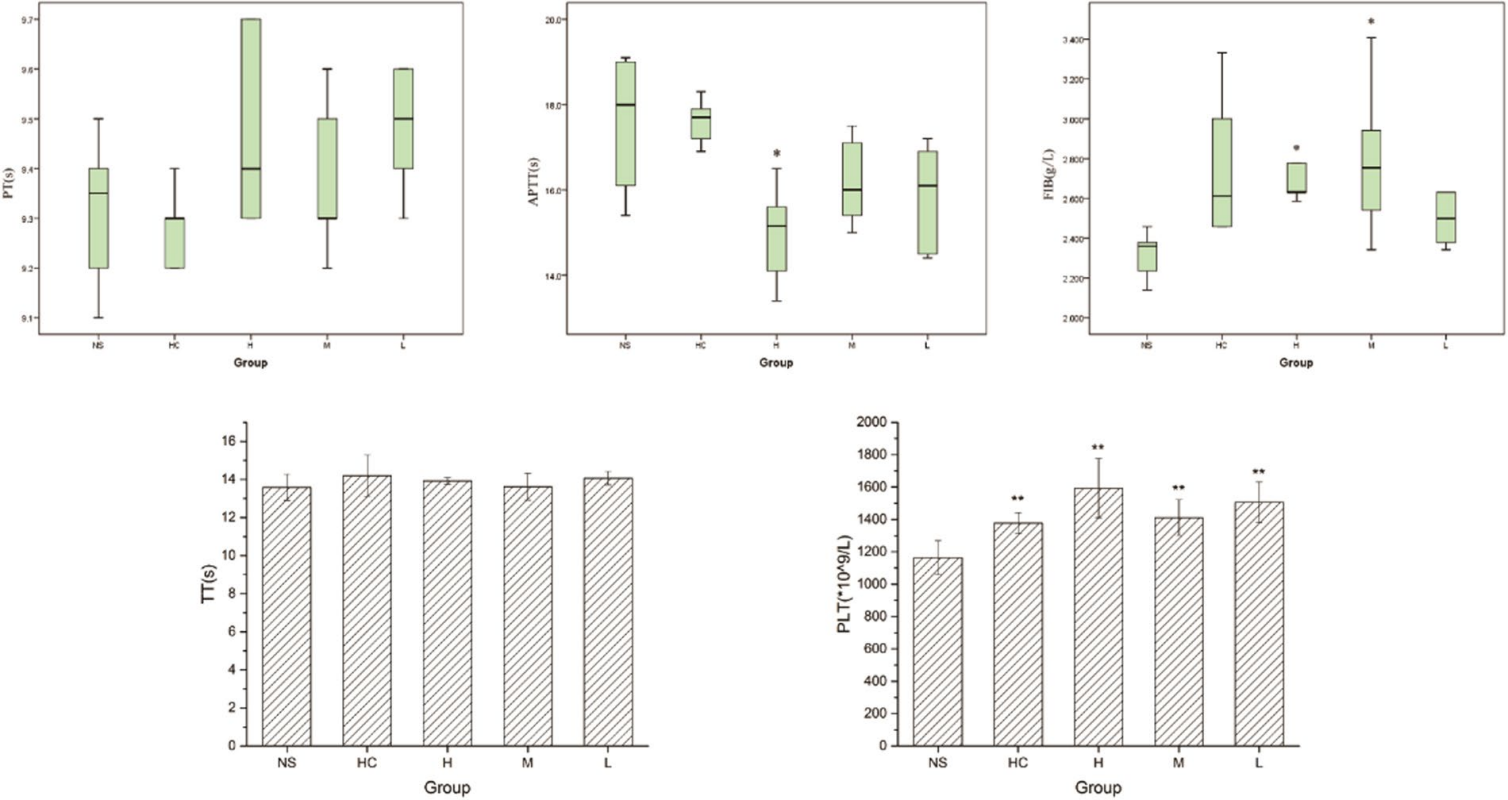
Haemostatic mechanism of egg yolk oil carbon quantum dots (EYO CDs) in mouse tail transection. The male Kunming mice were divided into normal group (treated with saline, NS), positive drug group (treated with haemocoagulase, HC), high-dose EYO CDs, medium-dose EYO CDs, and low-dose EYO CDs. (n = 9 per group). (**A**) The value of prothrombin time (PT) (**B**). The value of activated partial thromboplastin time (APTT) (**C**). The value of fibrinogen (FIB). (**D**)The value of thrombin time (TT) (**E**). The value of blood platelets (PLT).

As shown in Fig. 5, PT and TT values were not significantly different among the five treatment groups. The highest dose of CDs decreased APTT significantly (P < 0.05). Meanwhile, both the high and middle doses of CDs increased the FIB significantly (P < 0.05). All doses of CDs and the positive control drug increased the PLT significantly (P < 0.01), which is in agreement with the results of the bleeding times.

## Discussion

Based on previous research^23^, EYO contains benzo(a)pyrene (molecular weight 252.31)^24^, a fat-soluble carcinogen, which is difficult to remove from EYO. In this study, water extraction, ultrafiltration, and dialysis were used for the preparation of CDs and effectively reduced benzo(α)pyrene content to undetectable levels. This process will provide a solid foundation for developing future potential therapeutic medications.

Given that CDs present superior biocompatibility, low toxicity, decreased costs, robust chemical inertness, simple tunable emission, excitation-dependent emission properties, high photostability, non-blinking luminescence emission, excellent solubility, and easy functionalization^10^, they have attracted increasing attention from medical professionals. They are mainly used for fluorescence imaging and drug delivery^25^ in the medical field. Many CDs manufacturing methods have been developed, including pyrolysis^26^, hydrothermal^27^, calcination^28^, microwave assisted^29^, and laser-assisted^30^. Various raw materials have been adopted for the preparation of quantum dots, including glucose^31^, garlic^32^, wool^8^, candle soot^33^, and graphite^34^. This range of materials and techniques suggests that restrictions on what can be used for quantum dot production are limited only by the imagination.

Recently, varieties of new techniques and methods are reported for the synthesis and characterization of carbon nanoparticles^35, 36^. For instance, Manish Kumar *et al*. present a low temperature plasma sputtering process to deposit dense unhydrogenated carbon thin films^37^. Moreover, a key challenge in CDs formation is how to preserve stability of nanoparticles. In response, an advanced plasma process was presented to synthesize a nanocrystalline carbon films having highest values of surface energy which may enhanced the stability of nanoparticles^38^.

The results indicate that the main haemostatic mechanism of EYO CDs is stimulation of the intrinsic blood coagulation system and activation of the fibrinogen system. It is known that primary haemostasis involves a series of complex events initiated by injury of small blood vessels and culminating in arrest of bleeding. The blood clotting process includes the endogenous coagulation pathway (intrinsic) and exogenous coagulation pathway (extrinsic). By determining the value of the APTT, PT, TT, and FIB, we can evaluate the haemostatic effect and mechanism of EYO CDs. An effect on APTT and FIB, but not PT, suggests that the main haemostatic mechanism of EYO CDs is associated with the endogenous coagulation pathway (stimulating the intrinsic blood coagulation system and activating the fibrinogen system). However, this is only a preliminary explanation; the mechanisms for these effects still need further investigation. As CDs have a 5 nm particle size and a molecular weight of over 10 kD, the mechanisms of how CDs affect physiology is completely novel. The related studies and supporting data are limited. At present, the belief is that the surface groups may be the effective parts of the CDs, but more studies are warranted.

## Conclusions

In this study, a new material was isolated from EYO and identified as CDs. Additional pharmacodynamic experiments in mice revealed that the CDs exerted a dose-dependent effect on haemostasis. Coagulation assays suggested that EYO CDs stimulate the intrinsic blood coagulation system and activate the fibrinogen system. This study may provide novel strategies for studying the materials that form the foundations of traditional Chinese medicine.

## Electronic supplementary material

Supplementary Information
