# Supplementary material for: Novel carbon quantum dots from egg yolk oil and their haemostatic effects
Source: Sci Rep. 2017 Jun 30;7:4452. doi: 10.1038/s41598-017-04073-1 (PMC5493685; doi:10.1038/s41598-017-04073-1)
Supplement: Supplementary file 1 — Supplementary Information [file 41598_2017_4073_MOESM1_ESM.docx]

**Supplementary information for**

**Novel carbon** **quantum dots from egg yolk oil and their haemostatic effects**

Yan Zhao^1^, Yue Zhang^2^, Xiaoman Liu^1^, Hui Kong^2^, Yongzhi Wang^2^, Gaofeng Qin^1^, Peng Cao^1^, Xingxing Song^1^, Xin Yan^2^, Qingguo Wang^1^, Huihua Qu^3*^

*^1^School of Basic Medical Sciences, Beijing University of Chinese Medicine, Chaoyang, China*

*^2^School of Chinese Materia Medica, Beijing University of Chinese Medicine, Chaoyang, China*

*^3^* *Center of Scientific Experiment, Beijing University of Chinese Medicine, Chaoyang, China*

**
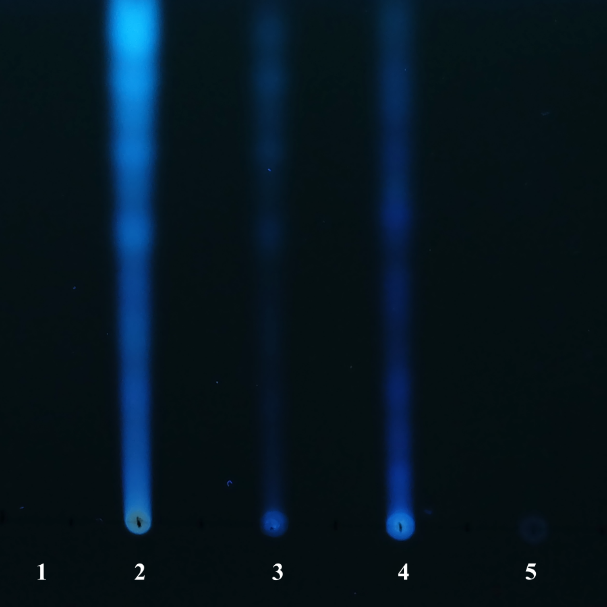
**

**Figure S1.** TLC of EYO-CDs at 365 nm. Lane 1 represents an aqueous solution of raw egg yolk powder, 2 represents a methanol solution of egg yolk oil, 3 represents EYO-CD solution before dialysis, 4 represents the dialysis solution from EYO-CD solution, and 5 represents the EYO-CD solution after dialysis.


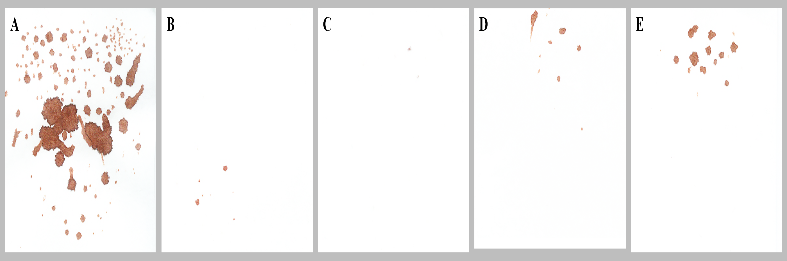


**Figure S2.** Haemostatic effect of EYO-CDs in mice tail transection. (A) Filter paper with normal group mice blood clots. (B) Filter paper with control group mice blood clots. (C) Filter paper with high dose group mice blood clots. (D) Filter paper with medium dose group mice blood clots. (E) Filter paper with low dose group mice blood clots.
